# Supplementary material for: Observation of Extraordinary Vibration Scatterings Induced by Strong Anharmonicity in Lead‐Free Halide Double Perovskites
Source: Adv Sci (Weinh). 2025 Jan 20;12(10):2408149. doi: 10.1002/advs.202408149 (PMC11905068; doi:10.1002/advs.202408149)
Supplement: Supplementary file 1 — Supporting Information [file ADVS-12-2408149-s001.docx]

**Supplementary information**

**Observation of Extraordinary Vibration Scatterings Induced by Strong Anharmonicity in Lead-Free Halide Double Perovskites**

Guang Wang^1#^, Jiongzhi Zheng^2#^, Jie Xue^3#^, Yixin Xu^1^, Qiye Zheng^1^, Geoffroy Hautier^2^, Haipeng Lu^3^ and Yanguang Zhou^^[[1]](#footnote-1)^*^

^1^*Department of Mechanical and Aerospace Engineering, The Hong Kong University of Science and Technology, Clear Water Bay, Kowloon, Hong Kong SAR, China*

*^2^Thayer School of Engineering, Dartmouth College, Hanover, New Hampshire 03755, USA*

*^3^Department of Chemistry, The Hong Kong University of Science and Technology, Clear Water Bay, Kowloon, Hong Kong SAR, China*

**Contents**

**Supplementary Note 1. The transducer P2**

**Supplementary Note 2. Sensitivity analysis P3**

**Supplementary Note 3. Cubic crystal structure of Cs_2_NaInCl_6_ P5**

**Supplementary Note 1. The transducer**

S 1.1 The thickness of the transducer

An Au film with a thickness of ~100nm was used as the transducer to enhance the thermal reflectance signal. In detail, a reference silica glass was covered with PI tape, and then was sent to the sputter chamber with sample. The thickness of Au for each batch of samples was measured by the atomic force microscope (AFM) after peeling off the PI tape. The tapping mode in AFM was used to scan across the step formed. As shown in **Figure S1**, the average thickness of Au for a typical sample is ~ 99 nm.


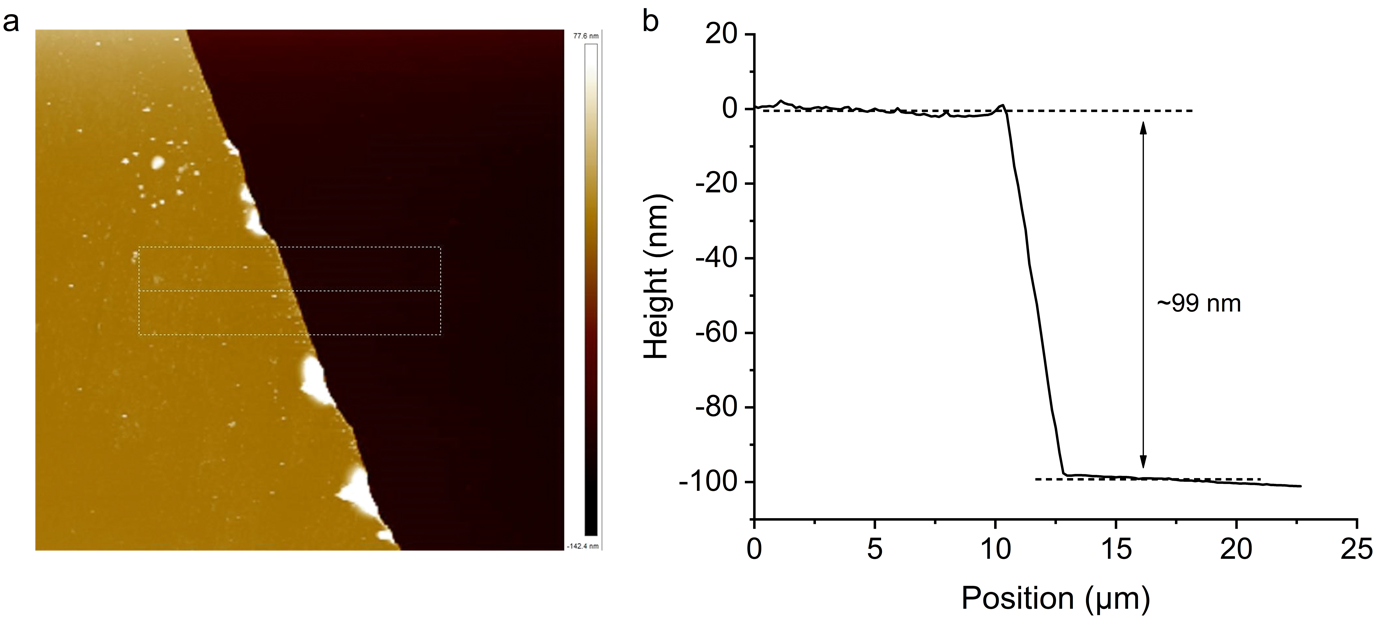
**Figure S1.** The thickness of the Au transducer. (a) The image of Au steps on reference silica. (b) The thickness of the region marked by the white dashed box in (a).

S 1.2 The thermal properties of Au transducer

To measure the temperature-dependent thermal conductivity of Cs_2_NaInCl_6_, the thermal conductivity and heat capacity of Au film at temperatures should be measured, as the thermal properties of thin Au film (~100 nm) are much different from bulk Au. We use FDTR to fit the thermal conductivity and heat capacity of Au film grown in fused silica reference sample. The temperature-dependent thermal properties of fused silica are derived from references. **Figure S2** shows the thermal conductivity and heat capacity of Au film obtained by FDTR measurements, which show an increasing trend with temperature and agree well with the references.


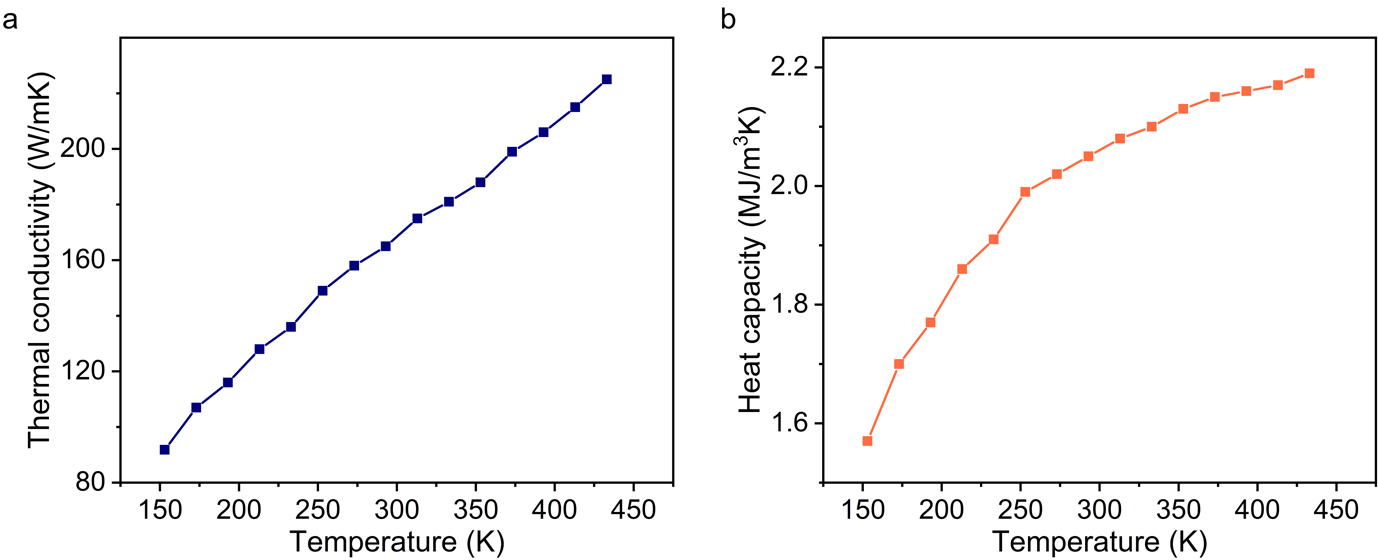


**Figure S2**. The thermal properties of a 100 nm Au film obtained by FDTR measurements. (a) The thermal conductivity and (b) the heat capacity at different temperatures.

**Supplementary Note 2. Sensitivity analysis**

We use sensitivity analysis to determine suitable fitting parameters and frequency range in FDTR fitting. It describes how sensitive a parameter is to the measurement by:

where $S(\omega)$ is the sensitivity, *x* is the interested parameter, and is the phase signal. We conducted the sensitivity analysis of the thermal conductivity, heat capacity and thermal conductance of Au/ Cs_2_NaInCl_6_ at room temperature, and the material properties used for sensitivity analysis were summarized in **Table S1**. We assumed the interested parameters were changed by a certain tolerance of 10%, the phase difference was shown in **Figure S3**. It is found that the heat capacity and thermal conductivity mostly overlapped in the high-frequency range, which is the most sensitive frequency for thermal conductivity. The sensitivity for interfacial thermal conductance (ITC) is much smaller than the other two. Therefore, we only fit the thermal conductivity of Cs_2_NaInCl_6_ in FDTR measurement. The heat capacity of Cs_2_NaInCl_6_ obtained by the first-principles calculation is used for fitting (**Figure S4**).


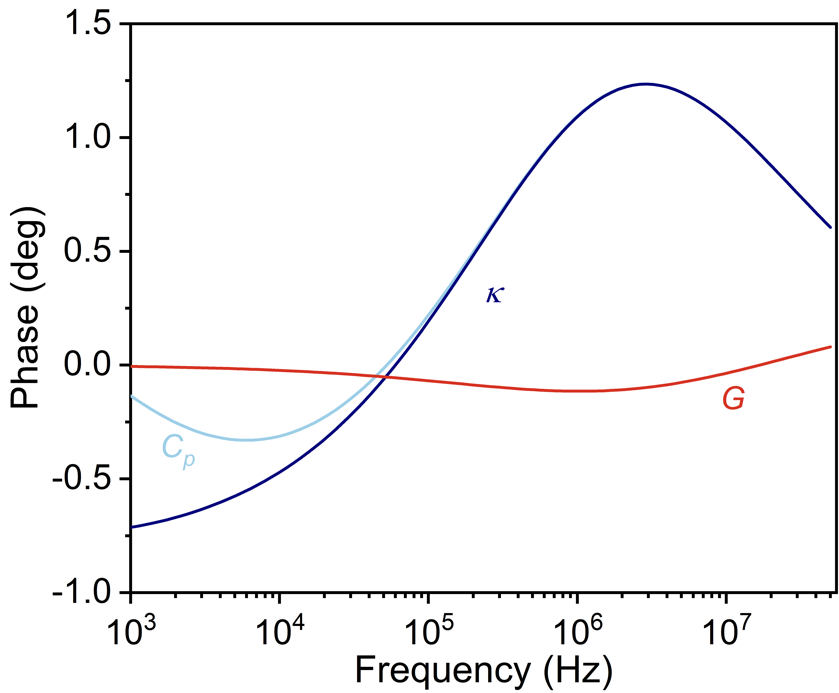


**Figure S3**. The sensitivity of Cs_2_NaInCl_6_’s thermal conductivity, heat capacity and ITC.

**Table S1**. Material properties that are needed for the sensitivity analysis at 300 K

| Materials | *C* ($10^{6}\text{ J m}^{\text{-3}}\text{K}^{\text{-1}}$) | $\kappa$ (Wm^-1^K^-1^) | *d* (nm) |
| --- | --- | --- | --- |
| Au | 2.05* | 165* | 100** |
| Cs_2_NaInCl_6_ | 1.37*** | 0.4/TBD | Semi-infinite |

*Obtained based on FDTR fitting, **obtained from AFM measurements, ***obtained by DFT calculation.


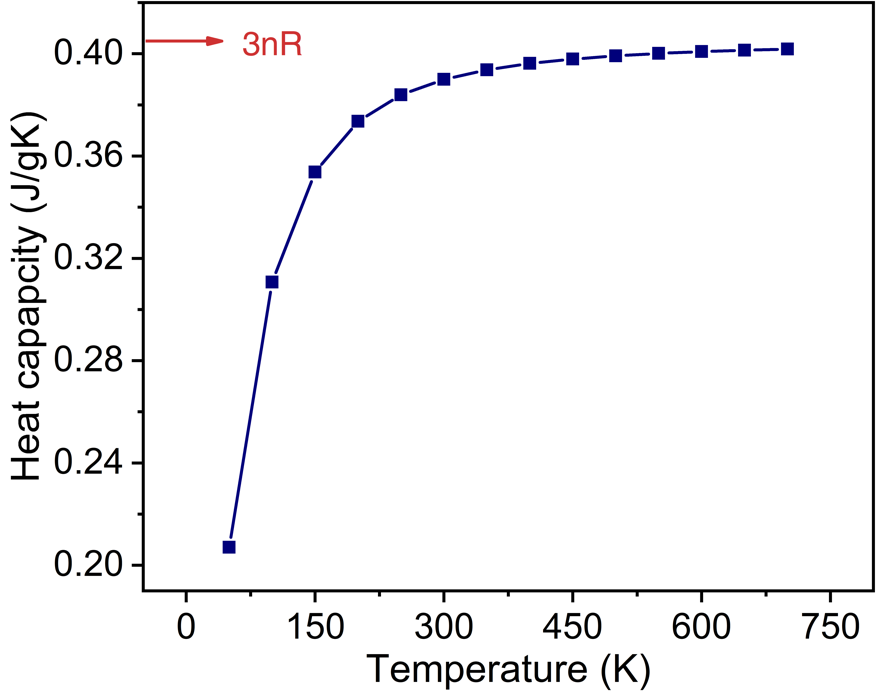


**Figure S4**. The specific heat capacity from the calculation, where *3nR* indicates the heat capacity’s Dulong-Petit limit.


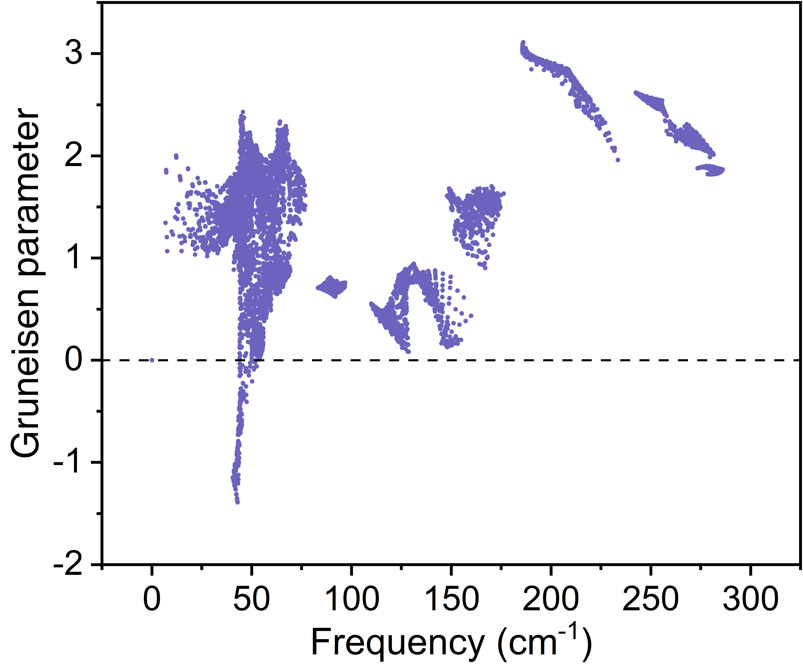


**Figure S5.** Modal Grüneisen parameters calculated by considering anharmonic phonon renormalization at 300 K.

**Supplementary Note 3. Cubic crystal structure of Cs_2_NaInCl_6_**

In this paper, we studied the thermal transport at different temperatures. It is true that many double perovskites have phase transitions at low temperatures. For example, Cs_2_AgBiBr_6_ transits from a tetragonal phase to a cubic phase at 122K (*1*).If Cs₂NaInCl₆ is tetragonal at low temperatures, imaginary frequencies will also exist in the phonon dispersion calculated based on static second-order force constants. However, our calculated phonon dispersion based on static second-order force constants of cubic Cs₂NaInCl₆ shows that there is no imaginary frequency (**Figure 5a**). Therefore, Cs₂NaInCl₆ should retain the cubic phase at low temperatures. This can also be reflected by the massive imagninary frequencies in the dispersion of hexagonal phase, as shown in **Figure S6**. Besides, we also run *ab initio* molecular dynamics simulations of cubic Cs₂NaInCl₆ in an *NPT* (number, pressure, and temperature) ensemble at several temperatures and find that the total energy has a linear relationship with temperature which indicates that there is no phase change (**Figure S7**). We further calculated the ground state energy of hexagonal and cubic Cs₂NaInCl₆. Our results show that the ground state energy of hexagonal (i.e. -2.96209735 eV/ atom) is larger than that of cubic (i.e. -2.9661420 eV/atom ) Cs₂NaInCl₆. The total energy calculated at higher temperature (200K) showed a higher energy of hexagonal phase (-469.5eV) than that of cubic phase (-555.6eV) as shown in **Figure S8**. Therefore, Cs₂NaInCl₆ should retain the cubic phase at the temperature range considered here. Furthermore, there is no abrupt change in our measured Raman at 4.4 K to 300 K, which indicates a stable cubic structure of Cs₂NaInCl₆. However, we emphasize that it is challenging to directly measure the corresponding heat capacity at lower temperatures due to the requirement of advanced facilities, which can directly show phase change. Nevertheless, our calculations and measurements show that Cs₂NaInCl₆ is cubic at the whole temperature range considered in this manuscript.


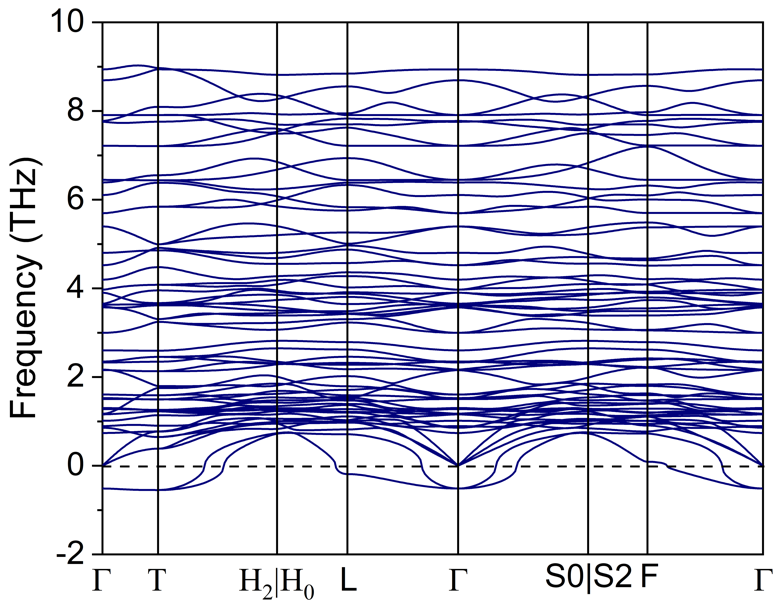


**Figure S6**. The phonon dispersion of hexagonal Cs2NaInCl6.


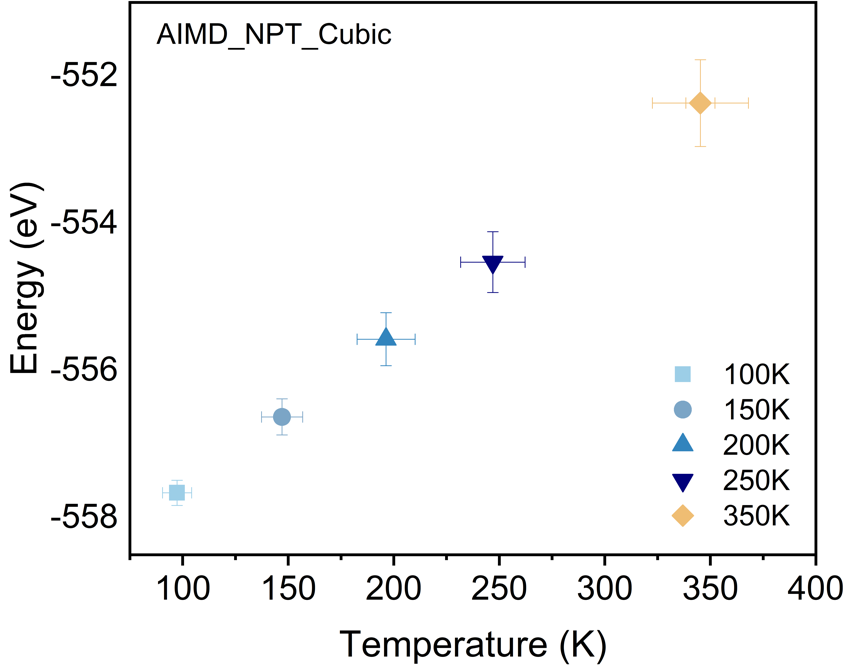


**Figure S7.** The total energy of the cubic phase of Cs₂NaInCl₆ at different temperatures calculated by AIMD.


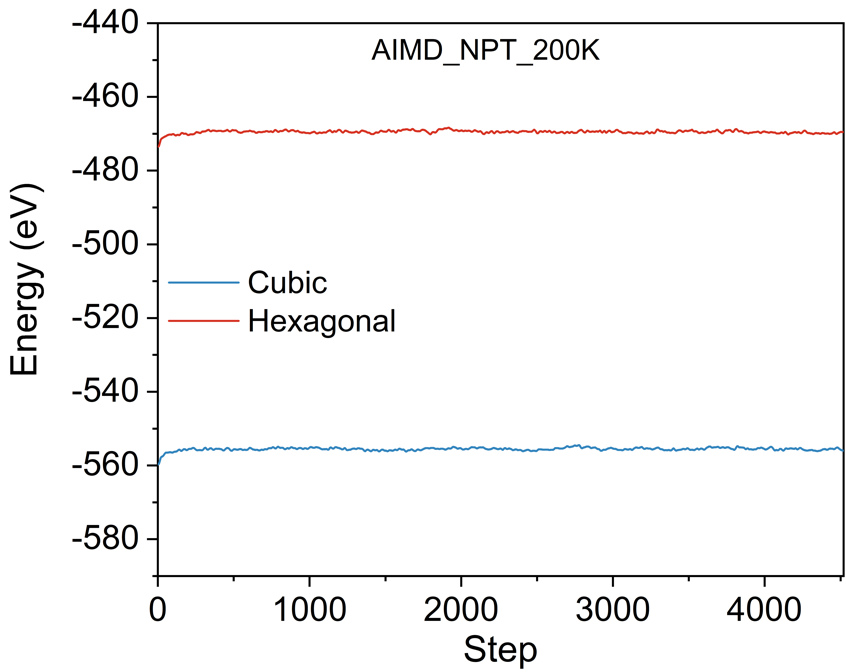


**Figure S8**. The total energy of hexagonal and cubic Cs_2_NaInCl_6_ calculated from AIMD at 200K.

**Table S2.** Anharmonic scores $\sigma^{A}$ from molecular dynamics and the measured thermal conductivities (*2*–*4*) for some typical crystals. The data in this table are used to plot **Figure 4a** in the main text.

| Materials | Types | $\sigma^{A}$ | $\kappa_{Exp}^{300K}$(W/mK) |
| --- | --- | --- | --- |
| Si | Zincblende | 0.151 | 165.6 |
| InP | Zincblende | 0.196 | 93 |
| CdTe | Zincblende | 0.325 | 7.5 |
| AgAlS_2_ | Tetragonal | 0.34 | 1.1 |
| LiInTe_2_ | Tetragonal | 0.345 | 1.08 |
| LiGaTe_2_ | Tetragonal | 0.32 | 0.8 |
| InTe | Tetragonal | 0.62 | 0.7 |
| AgAlSe2 | Tetragonal | 0.363 | 0.65 |
| TlSe | Tetragonal | 0.64 | 0.5 |
| AgTlI2 | Tetragonal | 1.21 | 0.25 |
| MgO | Rocksalt | 0.17 | 60 |
| MgO | Rocksalt | 0.17 | 55.2 |
| CaO | Rocksalt | 0.19 | 27 |
| LiF | Rocksalt | 0.33 | 17.6 |
| NaF | Rocksalt | 0.32 | 16.5 |
| LiH | Rocksalt | 0.3 | 14.7 |
| SrO | Rocksalt | 0.22 | 10 |
| KF | Rocksalt | 0.7 | 6.43 |
| NaCl | Rocksalt | 0.37 | 6 |
| NaBr | Rocksalt | 0.4 | 2.3 |
| BaO | Rocksalt | 0.5 | 2.3 |
| RbF | Rocksalt | 0.4 | 2.27 |
| PbSe | Rocksalt | 0.30251 | 2.2 |
| PbTe | Rocksalt | 0.445 | 2.2 |
| LiBr | Rocksalt | 0.7 | 1.83 |
| CuI | Rocksalt | 0.67 | 1.68 |
| NaI | Rocksalt | 0.43 | 1.33 |
| AgBr | Rocksalt | 0.94 | 1.1 |
| LiI | Rocksalt | 0.485 | 1.1 |
| SnSe | Rocksalt | 0.34 | 1 |
| AgCl | Rocksalt | 0.85 | 1 |
| AgCl | Rocksalt | 1.04 | 0.9 |
| CsF | Rocksalt | 0.478 | 0.83 |
| KMgF_3_ | Perovskite | 0.24 | 10 |
| SrTiO_3_ | Perovskite | 0.28 | 8.5 |
| KZnF_3_ | Perovskite | 0.32 | 5.5 |
| RbCaF_3_ | Perovskite | 0.49 | 3.4 |
| CsSnBr_3_ | Perovskite | 0.75 | 0.64 |
| CsSnI_3_ | Perovskite | 0.49 | 0.6 |
| GaN | Others | 0.151 | 210 |
| ZnO | Others | 0.243 | 60 |
| CdS | Others | 0.281 | 16 |
| CaF_2_ | Others | 0.31 | 9.76 |
| SrF_2_ | Others | 0.3 | 8.07 |
| CdF_2_ | Others | 0.42 | 4.3 |
| Mg_3_Sb_2_ | Others | 0.33 | 2.08 |
| KCaF_3_ | Others | 0.52 | 2 |
| KCdF_3_ | Others | 0.535 | 1.75 |
| AgGaSe_2_ | Others | 0.36 | 1 |
| Ti_3_VSe_4_ | Others | 0.3 | 0.3 |
| CNIC_50K | Cs_2_NaInCl_6_ | 0.238 | 1.4 |
| CNIC_300K | Cs_2_NaInCl_6_ | 0.389 | 0.43 |
| CNIC_400K | Cs_2_NaInCl_6_ | 0.421 | 0.366 |
| CABB_300K | Cs_2_AgBiBr_6_ | 0.827 | 0.35 |
| CABB_50K | Cs_2_AgBiBr_6_ | 1.38 | 0.8 |
| CsPbBr_3_ | CsPbBr_3_ | 0.66 | 0.42^(^*^5^*^)^ |
| CsPbCl_3_ | CsPbCl_3_ | 0.59 | 0.49^(^*^6^*^)^ |
|  |  |  |  |

# **References**

1. A. Cohen, T. M. Brenner, J. Klarbring, R. Sharma, D. H. Fabini, R. Korobko, P. K. Nayak, O. Hellman, O. Yaffe, Diverging Expressions of Anharmonicity in Halide Perovskites. *Advanced Materials* **34**, 2107932 (2022).

2. F. Knoop, T. A. R. Purcell, M. Scheffler, C. Carbogno, Anharmonicity in Thermal Insulators: An Analysis from First Principles. *Phys. Rev. Lett.* **130**, 236301 (2023).

3. Z. Zeng, X. Shen, R. Cheng, O. Perez, N. Ouyang, Z. Fan, P. Lemoine, B. Raveau, E. Guilmeau, Y. Chen, Pushing thermal conductivity to its lower limit in crystals with simple structures. *Nat Commun* **15**, 3007 (2024).

4. F. Knoop, T. A. R. Purcell, M. Scheffler, C. Carbogno, Anharmonicity measure for materials. *Phys. Rev. Materials* **4**, 083809 (2020).

5. W. Lee, H. Li, A. B. Wong, D. Zhang, M. Lai, Y. Yu, Q. Kong, E. Lin, J. J. Urban, J. C. Grossman, P. Yang, Ultralow thermal conductivity in all-inorganic halide perovskites. *Proceedings of the National Academy of Sciences* **114**, 8693–8697 (2017).

6. T. Haeger, M. Ketterer, J. Bahr, N. Pourdavoud, M. Runkel, R. Heiderhoff, T. Riedl, Thermal properties of CsPbCl3 thin films across phase transitions. *J. Phys. Mater.* **3**, 024004 (2020).

1. # These authors contribute equally. *Author to whom all correspondence should be addressed. Email: [maeygzhou@ust.hk](mailto:maeygzhou@ust.hk) [↑](#footnote-ref-1)
